# Effectiveness of a 12-Week Multi-Component Training Program with and without Transcranial Direct-Current Stimulation (tDCS) on Balance to Prevent Falls in Community-Dwelling Older Adults: A Study Protocol

**DOI:** 10.3390/biology11020290

**Published:** 2022-02-11

**Authors:** Laura Muñoz-Bermejo, Sabina Barrios-Fernandez, Jorge Carlos-Vivas, María Mendoza-Muñoz, Raquel Pastor-Cisneros, Eugenio Merellano-Navarro, Konstantinos Gianikellis, José Carmelo Adsuar

**Affiliations:** 1 Social Impact and Innovation in Health Research Group (InHEALTH), University of Extremadura, 06800 Mérida, Spain; 2 Promoting a Healthy Society Research Group (PHeSO), Faculty of Sport Sciences, University of Extremadura, 10003 Cáceres, Spain; jorgecv@unex.es (J.C.-V.); mamendozam@unex.es (M.M.-M.); raquelpc@unex.es (R.P.-C.); jadssal@unex.es (J.C.A.); 3 EFISAL Resarch Group, Universidad Autónoma de Chile, Talca 3460000, Chile; emerellanon@uautonoma.cl; 4 Biomecánica del Movimiento Humano y Ergonomía Research Group (BIOERGON), Faculty of Sport Sciences, University of Extremadura, 10003 Cáceres, Spain; kgiannik@unex.es

**Keywords:** balance, fall prevention, exercise, transcranial direct-current stimulation, physical fitness

## Abstract

**Simple Summary:**

Falls in community-dwelling individuals aged over 65 produce serious outcomes such as disability, morbidity, and mortality, as well as high healthcare costs. This research aims to assess whether a multicomponent training programme (McTP) combined with a transcranial direct-current stimulation device (tDCS), Halo Sport, produces improvements in balance and other gait-related parameters. Therefore, this study intends to test the efficacy of adding a tCDS device to an McTP in order to prevent falls in older adults by testing the safety, efficacy, and effectiveness of its implementation in care resources for the elderly.

**Abstract:**

Approximately one-third of elderly people aged over 65 who live in the community experience falls every year, with the proportion increasing with age. Moreover, of those who fall, about half will fall again in the following year. The falls’ consequences include disability, morbidity, and mortality. Although many external and internal factors lead to falls, balance issues play a major role. Multi-component training programs (McTP) usually combine balance, strength, cardiorespiratory fitness, and flexibility, with studies reporting multiple benefits on the health-related quality of life. Halo Sport is a transcranial direct-current stimulation (tDCS) device with promising results for gait performance. This study aims to test the effectiveness of the introduction of a tCDS device to an McTP to prevent falls in older adults. The sample will consist of 46 people aged 65 years or older, randomly assigned to experimental (n = 23) and control (n = 23) groups. The experimental group will perform the McTP while wearing tDCS, and the control group will perform McTP without the device, for three sessions per week over 12 weeks. The main measures will provide information about (1) safety, (2) applicability, (3) balance, (4) number of falls, (5) physical fitness, (6) risk of falling, (7) fear of falling, (8) health-related quality of life, and (9) cognitive function. Among the practical implications of this program, it is intended to provide data on its safety and effectiveness to be implemented in different resources as a tool for the prevention of falls.

## 1. Introduction

Ageing involves a series of complex changes that affect individuals in their physical, cognitive, emotional, and social areas. The biological mechanisms that cause ageing occur differently in each person, but the way in which a person ages depends not only on particular biological aspects but is also influenced by the person’s environment and behaviour, such that programs to address the health, quality of life and psychosocial aspects of older adults must be implemented [1]. Ageing is associated with changes in the endocrine, cardiovascular, respiratory, neurological, gastrointestinal and musculoskeletal and sensory systems [2], which may lead to a decline in functional capacity [3].

Falling is the result of a multiple-factor interaction that causes the individual to be unable to maintain or regain balance [4]. Falls are multifactorial; thus, biological, behavioural, social, economic, and environmental risk factors derived from community factors must be considered [5] (i.e., intrinsic and extrinsic factors [6]). A major contributor is balance, which can be defined as “a multidimensional concept, referring to the ability of a person not to fall” [7,8]. Estimates suggest that 13% of those aged 65–69, and up to 46% of those over 85, experience balance problems [9]. Balance is required in order to maintain a posture, perform voluntary movements, and react to external disturbances; individuals must be able to keep their centre of mass within their support base, with this skill being influenced by ageing [10].

Falls are frequent and particularly disabling [11], being one of the primary causes of mortality and morbidity in older adults [12,13]. Their consequences include fractures, bruising, lacerations, and sprains at a physical level [5], and a self-confidence loss and fear to fall again as common psychological symptoms, impacting the social and economic spheres, and affecting the quality of life [14]. Falls have become a serious public health problem because their consequences increase healthcare costs ($50 billion in the United States of America) [15].

Although falls are often considered to be part of the “normal ageing process”, they can be prevented [16]. Thus, multidisciplinary fall prevention programs should be implemented. Among the different types of interventions, exercise training has been shown to be effective [17,18,19]. However, the components of the programs influence the results. Different studies indicate that programs of which the main components were balance and functional tasks reduced falls rate by 24%, while those that included the categories included in the Prevention of Falls Network Europe taxonomy (ProFANE) [20], decreased the rate of falls by 34% [21]. The World Health Organization (WHO) [22] and the American College of Sports Medicine (ACSM) [23] recommend interventions that train physical capacities, recommending multi-component training programs (McTP) which involve strength, cardiorespiratory endurance, flexibility and balance training, as they have reported multiple improvements in functional capacity and health-related quality of life, and a lower rate of falls and cognitive impairment [24]. In the same line, the Spanish Ministry of Health recommends McTP that includes cardiopulmonary endurance, strength, power and functional capacity, flexibility, and balance training [25,26,27].

Non-invasive electrical brain stimulation (NIBS) is a promising and versatile technique used to study brain function and treat neurological and neuropsychiatric diseases [28]. Within the NIBS device typology, transcranial direct-current stimulation (tDCS) is a non-intrusive and painless brain stimulation technique that consists of the application of direct electrical currents to stimulate specific brain segments [29], differentiating two types of stimulation: anodal, to excite neuronal activity, and cathodal, to inhibit or reduce it [30]. Moreover, tDCS have generated great interest in areas such as cognitive functions and motor and physical performance [31,32]. The tDCS can stimulate neuroplasticity becase it modulates changes in neuronal membrane potential and produces an increase in cortical excitability [28]. Some works indicate that tDCS facilitates long-term potentiation by depolarizing the membrane potential, resulting in better motor learning, leading to possible gait performance improvements [33,34,35,36] and fall prevention. Halo Sport, a few years ago, marketed a device to improve physical performance based on tDCS [37]. It is a portable, easy-to-use, low-cost device consisting of a headset with an integrated tDCS headband that provides a weak direct current (DC) of below 2–3 milliampere (mA). It can be worn for up to 20 min. Surface electrodes, known as primers, are placed on the scalp to induce changes in both motor cortex sides [38], as they generate neural impulses for movement execution [39]. Halo Sport produces changes in motor cortex excitability, improving motor performance. One possible mechanism for this effect is that tDCS increases the intracortical facilitation of the motor cortex excitability, permitting these neurons to make new connections easily, improving skeletal muscle motor drive [40]. If gait performance is improved, benefits could include better mobility and a reduced fear of falling [41].

We aim to study whether the association of a multi-component training program (McTP) combined with transcranial direct-current stimulation (tDCS) device (Halo Sport) is safe, applicable, and can enhance performance on gait and balance. Additionally, this project has a secondary aim to evaluate the effect of the combined program on the falls, physical fitness, health-related quality of life, and cognitive function of participants, and the safety and applicability of the program. We hypothesize that the group that performs the McTP combined with tDCS will obtain better results in the primary variable, balance, as well as in the secondary variables.

## 2. Materials and Methods

### 2.1. Study Design

A randomized controlled trial will be conducted with a 1:1 assignment ratio between the experimental and control groups. The Consolidated Standards for Reporting Trials will be followed (CONSORT) [42].

### 2.2. Ethics and Registration

This study has been approved by the Bioethics and Biosafety Committee of the University of Extremadura (approval number: 184/2021). Moreover, the study has been registered in the Clinical Trials Registry provided by the Australian New Zealand Clinical Trial Registry (Request number: 383356; https://www.anzctr.org.au/ (accessed on 9 October 2021)).

### 2.3. Sample Size

The sample size was estimated based on the Time Up and Go (TUG) test results in older people, a test which is considered valid and reliable for this population [43,44]. Thus, accepting a risk alpha of 0.05 and a risk beta of 0.2 in a bilateral contrast, a total of 44 individuals (22 both in the experimental and control group) are needed in order to find a difference equal to or higher than 0.97 units. A common standard deviation of 1.3 and a correlation coefficient between the baseline and end-line measurement of 0.7 are assumed [45]. A loss of follow-up rate of 20% is predicted.

### 2.4. Randomization and Blinding

After the initial assessment, a simple computer-generated randomisation sequence will be generated to assign the participants (1:1) to the experimental (McTP + tDCS) and control (McTP) groups using Research Randomizer software (Version 4.0, Geoffrey C. Urbaniak y Scott Plous, Middletown, CT, USA; http://www.randomizer.org, accessed on 15 September 2021) [46]. A researcher not involved in the study will prepare the randomization sequence. This information will be hidden in a password-protected file. Both the participants and professionals who will lead the sessions will be aware of their group allocation; however, it will be hidden from the data analysis investigators.

### 2.5. Eligibility Criteria

The participants will comply with the following inclusion criteria: (a) age ≥ 65 years old; (b) not suffering any pathology that contraindicates physical activity(the Physical Activity and Fitness Questionnaire (PAR-Q) will be run) [47]; (c) not presenting signs of cognitive impairment based on the Mini-Mental State Examination (scores > 25) [48]; (d) not presenting contraindications to tDCS use; (e) participants must provide to the researchers a signed informed consent form; and (f) they must show motivation to participate in the program. Moreover, the exclusion criteria will include: (a) living in a nursing home; (b) needing an assistive device to move or walk; (c) having any of the lower limbs amputated; or (d) presenting sensorial impairment that makes the normal development of exercise difficult.

### 2.6. Intervention

#### 2.6.1. The Multicomponent Exercise Program

Before starting the first training session, the participants will receive an explanation of the protocol to follow. The program will be composed of 3 sessions (30 min) per week over 12 weeks. Each session will be supervised by experts in the field. The structure of the sessions will consist of a 10-min warm-up (joint mobility, stretching), a 20-min main part of McTP exercises or activities wearing the Halo, and a 10-min cool-down (stretching, final thoughts and personal hygiene). The participants will wear the Halo only during the main part (20 min). The McTP will consist of balance, strength, cardiorespiratory endurance, and flexibility exercises, with several of these components being trained in each session. Examples of the activities are provided in Table 1. Furthermore, each participant will be asked for the Rating of Perceived Exertion [49] at the end of each session in order to monitor the intensity of the session.

The McTP includes gradual increases in exercise intensity, volume and complexity, together with accompanying balance, strength, endurance and flexibility exercises [25]. 

#### 2.6.2. Experimental Group

The experimental group will perform the McTP while wearing the Halo Sport (Halo Neuroscience, San Francisco, CA, USA; https://www.haloneuro.com, accessed on 9 October 2021), a commercial tDCS device which is similar to audio headphones. Three-pointed foam electrodes (24 cm^2^/primer), moistened before use, will make electrical contact with the head. The Halo Sport will be placed on the vertex of the head after cleaning the stimulation area with alcohol swabs, and the integrated electrodes of the device will be saturated with water. The primers are located on the upper head to stimulate both sides of the motor cortex. The electrodes are linked to a DC electrical stimulator, powered by a lithium-ion cell (LiPo 36 V). The anodal electrode is positioned on the top of the head, and the cathodal electrodes on the left and right sides. The current of the anodal tDCS will be set at 2 mA for 20 min [50], and will be monitored by the Halo Sport app in a mobile device [51,52]. The participants will remain seated in a resting state while the Halo Sport headphones are placed on them, and will perform the exercises while equipped with the device. 

#### 2.6.3. Control Group

Participants will perform the McTP program without the tDCS stimulation. 

Both the experimental and control groups will participate in the initial, mid-term and final evaluations.

### 2.7. Measures and Procedures

Before taking any measurements, a test familiarisation session will be conducted with the participants. A set of evaluation tests will be applied to evaluate the program’s usefulness and effectiveness through initial, mid-term and final assessments, as shown in Table 2.

#### 2.7.1. Primary Measures

Balance will be assessed using the TUG [43,44], and the Activity-Specific Balance Confidence Scale (ABC) [7,53,54] will be performed. The 3-metre variant of the TUG will be performed. The participant, sitting in a chair, with arms and trunk resting on the backrest, stands up on the investigator’s signal and walks in a straight line, turns 180 degrees, walks back, and sits back down in the chair as fast as possible. Before recording the measurements (there will be two attempts with a one-minute break), a familiarisation test will be performed. The time will be measured from the start of the standing movement until the participant sits down again with his/her back against the backrest. The data from the best attempt will be taken for analysis. The ABC questionnaire is a 16-question tool that assesses the confidence a person has in their balance when carrying out daily activities, with scores ranging from 0 to 100. A score of more than 67% is considered to be a high risk of falling.

#### 2.7.2. Secondary Measures

Socio-demographic data: The participants will complete a questionnaire about their age, gender, educational level, marital status, living unit and health information.

Risk of falling: The Fall Risk Index questionnaire (FRI-21) will be administered. It is a 21-item instrument that assesses both the internal and external factors present in the risk of falling. Each item is scored as 1 (risk) or 0 (no risk), with higher scores implying a greater falls risk. A 9 to 10 cut-off point is considered suitable for the early detection of fall risk [55].

Fear of falling: The Falls Efficacy Scale-International (FES-I), developed by the ProFaNE, will be applied. It is a 16-item self-report instrument that collects information on concerns about falls in a variety of daily activities, with excellent reliability and validity values (intraclass correlation coefficient = 0.96) [56,57]. Its score consists of a 4-point scale (from 1, “slightly worried”, to 4, “very worried”).

Number of falls: The number of falls suffered by participants in the last three and six months, and the last year, will be recorded. However, only the outcomes for the last three months will be used to analyze the effect of the intervention, while the falls in the last six months and the last year will only be used to characterise the sample.

Physical fitness:

- The 2-min walk test assesses the longest distance in metres that a person can walk along a rectangular path. It has high reliability, and its intraclass correlation coefficient is 0.888 [58].
- Lower body strength: The 30-s Chair Stand Test will be performed, which consists of sitting down and standing up from a chair for 30 s, counting the repetitions that the person can perform. Its reliability is considered high (0.87) [59].
- Lower limb flexibility: This will be measured by performing the Sit and Reach Test. Participants will sit with one leg extended and then slowly bend over, sliding their hands down the extended leg trying to touch (or pass) the toes of the toe line. The number of centimetres before reaching (negative score) or beyond (positive score) the toe will be recorded [60]. Two attempts will be measured for each leg. The average of the best results of both legs will be considered for the analysis. Its intraclass correlation coefficient value is 0.92, which shows high reliability [61].
- Speed: The Brisk Walking Test will be performed. The time taken to cover 30 m walking will be considered. Two repetitions will be conducted, with a one-minute rest between them [62], taking the best repetition for the analyses. The test–retest reproducibility of this assessment is 0.95, and its Cronbach’s alpha coefficient of reproducibility is 0.96 [63].
- The Short Physical Performance Battery (SPPB) consists of three direct observation tasks: walking speed, balance, and the time taken to get up five times from a chair [64]. The Cronbach’s alpha value is 0.70 [65].
- Self-perception of physical fitness: The International Fitness Scale (IFIS) [66] will be administered. It permits us to obtain information about the participants’ general physical fitness self-perception, as well as their cardiorespiratory fitness, strength, speed-agility, and flexibility. This scale is composed of five questions with five response options (“very bad”, “bad”, “average”, “good” and “very good”).

Health-related quality of life: The Short Form-12 (SF-12) questionnaire will be used. This tool has eight elements (physical function, physical role, bodily pain, general health, vitality, social function, emotional role, and mental health), and two short components, physical and mental. The score ranges from 0 to 100 (0 being the worst and 100 being the best status). The SF-12 allows us to obtain the quality-adjusted life-years of life SF-6D [67,68].

Cognitive function: The Montreal Cognitive Assessment (MoCA) will be applied. It is a screening test for cognitive impairment that assesses eight cognitive domains (attention, executive functions, numeracy, language, working memory and recall, abstraction, orientation, and visuospatial processing) [69].

Safety: A log will be kept for each of the sessions in which any incident, injury or problem that arises will be noted, recording the possible origin of the problem.

Applicability: The percentage of participants who carry out the programmed activities will be calculated. If an individual is not able to carry out the activities, the reason will be indicated.

### 2.8. Statistical Analysis

The statistical analysis will be conducted through the Statistical Package for the Social Sciences software (SPSS, Version 25, IBM SPSS, Armonk, NY, USA).

The characteristics of the study participants will be presented as the mean (standard deviation) and proportions for continuous and categorical variables, respectively. We will carry out an intention-to-treat (all participants), and a per-protocol analysis (individuals who complete the program).

Intention-to-treat analysis: All of the randomised participants will be considered for the analysis in their relevant group. Missing data will be imputed through multiple imputations. Repeated measures analyses of covariance (ANCOVA) adjusted by age and baseline outcomes will be applied to analyse the intervention effects on the different dependent variables. The baseline outcomes will be applied as a covariate for all of the dependent variables’ pre–post-comparisons in order to avoid the bias of possible differences between the groups in the initial outcomes. The effect sizes (95% confidence interval) and differences for each variable concerning the time and the group × time interaction will be calculated. The alpha level will be set at *p* < 0.05. The data imputation for the sensitivity analyses will be conducted on patients who presented data at the baseline, and who completed the study, in order to avoid estimation bias.

Per-protocol analysis: The analyses previously described will be carried out but considering only those participants who attended at least 75% of the program sessions.

## 3. Discussion

This study the efficacy of Halo-implemented McTP to improve balance, in order to improve postural stability to decrease falls. Moreover, it presents direct measures of safety and applicability (Halo Sport) for its applications in community-dwelling older adults, providing data about their effectiveness in the prevention of falls, functional capacity, and quality of life. This is a novel approach starting from an integrative perspective, including elements that combine physical motivation and cortical stimulation.

The high incidence of falls in the elderly [70] underlines the need to boost the design of prevention programs. The impact of frailty in older people constitutes a prominent health problem. The decline in muscle mass associated with age has a negative influence on strength and movement capacity, leading to a reduced quality of life [71]. Muscle strength and balance are, therefore, key components of physical activity programs to reduce the fall risk in the elderly [72]. This program complies with the recommendations from the WHO [22] and the ACSM [23] by taking into account strength, cardiorespiratory endurance, balance and flexibility.

In addition, the results obtained with tDCS techniques—which have shown an improvement in motor activity, locomotion, mobility and gait speed [73,74,75]—are in the same line. If the effectiveness of the Halo-implemented McTP on motor learning and improvement of balance, fitness and lower-limb strength is demonstrated, conventional training programmes could be implemented with this kind of device to decrease the risk of falls. Furthermore, it would have an impact on the sociosanitary area through its possible implementation in primary care, rehabilitation and care centres in order to reduce the number of falls. If positive outcomes are obtained, the intervention proposed could be offered as an alternative or complementary tool for health professionals to enhance the quality of life of the elderly, and to minimise the economic impact of falls.

## 4. Conclusions

This trial will research Halo-implemented McTP’s efficacy, safety and applicability to increase balance in order to prevent falls in community-dwelling older adults. We hypothesise that the combined program (McTP-tDCS) will be more effective than the McTP carried out in isolation.

## Figures and Tables

**Table 1:** Examples of the activities and exercises performed according to the physical abilities in the multi-component training programme.

Physical Ability	Examples
Balance	Exercises on one foot, tandem or semi-tandem position. Exercises in movement walking with heel to toe support. Line walking. Multidirectional movements with extra weights. Altering the base of support. Modified Tai Chi exercises.
Strength	Free weights. weight-bearing exercises. Machines exercises. Pilates.
Cardiorespiratory endurance	Walking indoors and outdoors. Treadmill walking. Stationary cling. Climbing steps/stairs.
Flexibility	Stretches. Yoga.

**Table 2:** Schedule of the evaluations in the experimental and control groups.

Measures	Week 0	Week 6	Week 12
Sociodemographic data	√		
Balance	√	√	√
Number of falls (a) last year (b) last 6 and (c) 3 months	√	√	√
Physical fitness	√	√	√
Fall Risk	√	√	√
Fear of falling	√	√	√
Health-related quality of life	√	√	√
Cognitive function	√	√	√
Security	√		√
Applicability	√		√

## Data Availability

The datasets used during the current study are available from the corresponding authors on reasonable request.
